# Posterior Tracheopexy for Severe Tracheomalacia Associated with Esophageal Atresia (EA): Primary Treatment at the Time of Initial EA Repair versus Secondary Treatment

**DOI:** 10.3389/fsurg.2017.00080

**Published:** 2018-01-15

**Authors:** Hester F. Shieh, C. Jason Smithers, Thomas E. Hamilton, David Zurakowski, Gary A. Visner, Michael A. Manfredi, Christopher W. Baird, Russell W. Jennings

**Affiliations:** ^1^Department of Surgery, Boston Children’s Hospital, Harvard Medical School, Boston, MA, United States; ^2^Department of Pulmonology, Boston Children’s Hospital, Harvard Medical School, Boston, MA, United States; ^3^Department of Gastroenterology, Boston Children’s Hospital, Harvard Medical School, Boston, MA, United States; ^4^Department of Cardiovascular Surgery, Boston Children’s Hospital, Harvard Medical School, Boston, MA, United States

**Keywords:** posterior tracheopexy, tracheomalacia, esophageal atresia, brief resolved unexplained events, airway collapse

## Abstract

**Purpose:**

We review outcomes of posterior tracheopexy for tracheomalacia in esophageal atresia (EA) patients, comparing primary treatment at the time of initial EA repair versus secondary treatment.

**Methods:**

All EA patients who underwent posterior tracheopexy from October 2012 to September 2016 were retrospectively reviewed. Clinical symptoms, tracheomalacia scores, and persistent airway intrusion were collected. Indication for posterior tracheopexy was the presence of clinical symptoms, in combination with severe tracheomalacia as identified on bronchoscopic evaluation, typically defined as coaptation in one or more regions of the trachea. Secondary cases were usually those with chronic respiratory symptoms who underwent bronchoscopic evaluation, whereas primary cases were those found to have severe tracheomalacia on routine preoperative dynamic tracheobronchoscopy at the time of initial EA repair.

**Results:**

A total of 118 patients underwent posterior tracheopexy: 18 (15%) primary versus 100 (85%) secondary cases. Median (interquartile range) age was 2 months (1–4 months) for primary (22% type C) and 18 months (8–40 months) for secondary (87% type C) cases (*p* < 0.001). There were statistically significant improvements in most clinical symptoms postoperatively for primary and secondary cases, with no significant differences in any postoperative symptoms between the two groups (*p* > 0.1). Total tracheomalacia scores improved significantly in primary (*p* = 0.013) and secondary (*p* < 0.001) cases. Multivariable Cox regression analysis indicated no differences in persistent airway intrusion requiring reoperation between primary and secondary tracheopexy adjusting for imbalances in age and EA type (*p* = 0.67).

**Conclusion:**

Posterior tracheopexy is effective in treating severe tracheomalacia with significant improvements in clinical symptoms and degree of airway collapse on bronchoscopy. With no significant differences in outcomes between primary and secondary treatment, posterior tracheopexy should be selectively considered at the time of initial EA repair.

## Introduction

Tracheomalacia is often associated with esophageal atresia (EA), tracheoesophageal fistula (TEF), and cardiac disease (1, 2). Severe tracheomalacia is characterized by dynamic airway collapse in spontaneously breathing patients with anterior vascular compression, posterior membranous tracheal intrusion, or both (3). Aortopexy addresses anterior vascular compression by indirectly elevating the anterior wall of the trachea but does not directly address posterior membranous tracheal intrusion (4). We previously reported a series of patients who underwent posterior tracheopexy for severe tracheomalacia with posterior intrusion with promising short-term results, although these reports did not distinguish between primary treatment at the time of initial EA repair and secondary treatment (2, 3, 5). We now review outcomes of posterior tracheopexy in EA patients, comparing primary treatment at the time of initial EA repair versus secondary treatment, to determine whether there were resolution of clinical symptoms and bronchoscopic evidence of improvement in airway collapse.

## Materials and Methods

The Esophageal and Airway Treatment (EAT) Center at Boston Children’s Hospital is a multidisciplinary care team consisting of three pediatric surgeons, one pediatric cardiothoracic surgeon, one pediatric pulmonologist, and two pediatric gastroenterologists. We retrospectively reviewed all EA patients who underwent posterior tracheopexy at Boston Children’s Hospital from October 2012 to September 2016 under an approved institutional review board protocol (IRB-P00021702). Primary treatment patients underwent posterior tracheopexy at the time of initial EA repair, whereas secondary patients underwent posterior tracheopexy after prior esophageal surgery. Prior surgery included thoracotomy with primary EA repair, repair of proximal or distal TEF, EA repair by Foker process, and esophageal replacement.

Patient demographics, pre- and postoperative clinical symptoms and airway evaluation, surgical techniques, and persistent airway intrusion requiring reoperation, were collected. Patients were evaluated by the EAT team for the presence of clinical symptoms including cough, barking cough, noisy breathing, prolonged and recurrent pulmonary infections, exercise intolerance, transient respiratory distress requiring positive pressure, oxygen and ventilator dependence, blue spells, and brief resolved unexplained events (BRUEs).

Pre- and postoperative endoscopic airway evaluation was performed by the primary surgeons. Diagnostic laryngoscopy and bronchoscopy was done under general anesthesia in spontaneously breathing patients to assess supraglottic structures and vocal cord function, as well as dynamic motion in the tracheobronchial tree throughout the respiratory cycle, then heavily sedated to evaluate the larynx for presence of a laryngeal cleft, and the presence of TEFs or tracheal diverticula. A standardized tracheomalacia scoring system based on dynamic airway evaluation was used to determine pre- and postoperative tracheomalacia scores (Table 2) (3, 5, 6). The degree of open airway was scored out of 100 at each anatomic region: upper (T1), middle (T2), and lower (T3) trachea, and right and left mainstem bronchi, with a maximum score of 500. Severe tracheomalacia was typically defined as coaptation in one or more regions of the trachea. Dynamic airway multidetector computed tomography was performed at surgeon discretion to evaluate for aberrant vascular anatomy or associated lung parenchymal disease for operative planning (6). Indication for posterior tracheopexy was the presence of clinical symptoms, in combination with severe tracheomalacia as identified on bronchoscopic evaluation.

Generally patients with EA underwent right posterior thoracotomy in those with a left sided aortic arch, whereas those with associated cardiac disease underwent sternotomy. The esophagus, back wall of the trachea, thoracic duct, and/or aorta were fully dissected and mobilized, taking care to protect the left vagus nerve and left recurrent laryngeal nerve. A recurrent TEF or residual tracheal diverticulum from a previously repaired TEF was corrected if present by resecting the TEF or diverticulum flush with the tracheal wall under bronchoscopic visualization. Posterior tracheopexy was performed by passing autologous pledgeted polypropylene sutures into but not through the posterior tracheal membrane, and securing them to the anterior longitudinal spinal ligament under direct bronchoscopic guidance.

Categorical data are expressed as percentages with Fisher’s exact test used to assess primary and secondary group differences. Continuous data are summarized using median and interquartile range (IQR), with groups compared by the nonparametric Mann-Whitney U-test. To assess resolution of clinical symptoms, the percentage of patients with each symptom pre- and postoperatively was compared by the Wald chi-square test using logistic regression modeling with generalized estimating equations to account for the binary paired data. Changes in tracheomalacia scores for each airway segment were determined by the Wilcoxon signed-ranks test. Multivariable Cox proportional hazards regression analysis was performed to identify independent predictors of time to persistent airway intrusion requiring reoperation while adjusting for covariates. Statistical analysis was performed using SPSS Statistics (version 23.0, IBM Corporation, Armonk, NY, USA). A two-tailed *p*–value <0.05 was statistically significant.

## Results

A total of 118 EA patients underwent posterior tracheopexy at median age 16 months (IQR 5–32 months): 18 patients (15%) in the primary group and 100 patients (85%) in the secondary group. There were no significant differences in sex, gestational age, cardiac disease, and VACTERL syndrome between primary and secondary groups (Table 1). Primary patients consisted of significantly more type A (44 vs. 9%, *p* < 0.001) and long gap (72 vs. 29%, *p* < 0.001) EA, while secondary patients were those with a prior type C repair (87 vs. 22%, *p* < 0.001). 8% of the secondary group had a prior aortopexy. Primary patients underwent tracheopexy at a significantly younger age than secondary patients, median age 2 months (IQR 1–4 months) vs. 18 months (IQR 8–40 months) (*p* < 0.001).

**Table 1:** Demographics.

Variable	Primary (*n* = 18)	Secondary (*n* = 100)	*p*-Value
Sex (% male)	44	52	0.56
Estimated gestational age (weeks)	36 (32–38)	35 (33–37)	0.90
Esophageal atresia			
– Type A	44%	9%	<0.001*
– Type B	22%	3%	0.001*
– Type C	22%	87%	<0.001*
– Type H	11%	1%	0.01*
– Long Gap	72%	29%	<0.001*
– Associated TEF	56%	93%	<0.001*
Cardiac disease	44%	30%	0.23
VACTERL	33%	32%	0.91
Age at tracheopexy (months)	2 (1–4)	18 (8–40)	<0.001*

*Continuous data are median (IQR)*.

*Indicating statistical significance.

Upper airway anomalies were common. 3% had laryngomalacia and 4% had preoperative left vocal cord paralysis. 21% had laryngeal clefts: 14% type 1, 5% type 2, 2% type 3, and 1% type 4. 11% had subglottic stenosis. There were no significant differences in preoperative tracheomalacia scores between primary and secondary groups (Table 2). The middle (T2) and lower (T3) trachea were the most severely affected preoperatively, with overall median scores of 0 (IQR 0–25) at each region.

**Table 2:** Tracheomalacia scores.

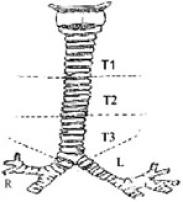	Location	Preoperative (*n* = 118)	Postoperative (*n* = 76)	*p*-Value
	T1	80 (70–100)	100 (80–100)	<0.001*
	T2	0 (0–25)	80 (60–100)	<0.001*
	T3	0 (0–25)	100 (52–100)	<0.001*
	Right bronchus	100 (80–100)	100 (100–100)	<0.001*
	Left bronchus	100 (50–100)	100 (92–100)	0.002*
	Total	270 (220–320)	440 (390–495)	<0.001*

Primary (*n* = 18)	Location	Preoperative (*n* = 18)	Postoperative (*n* = 15)	*p*-Value
	T1	80 (50–100)	90 (80–100)	0.342
	T2	22 (0–45)	75 (50–100)	0.010*
	T3	20 (0–40)	75 (40–100)	0.014*
	Right bronchus	100 (98–100)	100 (100–100)	0.833
	Left bronchus	100 (50–100)	100 (50–100)	0.887
	Total	300 (242–342)	400 (325–460)	0.013*

Secondary (*n* = 100)	Location	Preoperative (*n* = 100)	Postoperative (*n* = 61)	*p*-Value
	T1	80 (70–98)	100 (80–100)	<0.001*
	T2	0 (0–20)	80 (65–100)	<0.001*
	T3	0 (0–20)	100 (60–100)	<0.001*
	Right bronchus	100 (76–100)	100 (100–100)	0.004*
	Left bronchus	90 (50–100)	100 (100–100)	0.001*
	Total	260 (216–320)	450 (390–500)	<0.001*

*Pre- and postoperative tracheomalacia scores based on standardized bronchoscopic evaluation for the overall cohort, as well as primary and secondary groups. Scores are percentage of open airway out of 100 for each anatomical region. Data are median (IQR)*.

*Indicating statistical significance.

Operative approach was primarily by right thoracotomy in 85% of patients. Other approaches included median sternotomy in 3%, left thoracotomy in 2%, left neck dissection in 1%, and combined neck and chest approaches in 9%. All patients underwent posterior tracheopexy under intraoperative bronchoscopic guidance. 20% underwent additional procedures to open their airway, including anterior aortopexy in 2%, anterior tracheopexy in 3%, descending posterior aortopexy in 14%, left or right mainstem bronchopexy in 9%, pulmonary artery pexy in 1%, and innominate artery pexy in 2%. 5% had an aberrant right subclavian artery behind the trachea, requiring mobilization of the artery in three patients, division of the artery in one patient, and translocation/reimplantation to the carotid artery or ascending aorta in the other two patients. 67% had an associated tracheal diverticulum that was resected flush with the trachea.

Primary patients, as compared to secondary patients, had significantly longer postoperative ventilator days [median (IQR) 20 (6–24) vs. 4 (1–7) days, *p* < 0.001], intensive care unit stay [median (IQR) 28 (16–46) days vs. 7 (2–16) days, *p* < 0.001], and hospital length of stay [median (IQR) 50 (22–100) vs. 15 (7–37) days, *p* < 0.001], generally related to the esophageal repairs, especially children requiring Foker process. There were no significant early complications including hemorrhage or infection. There were no mortalities.

**Figure 1:**
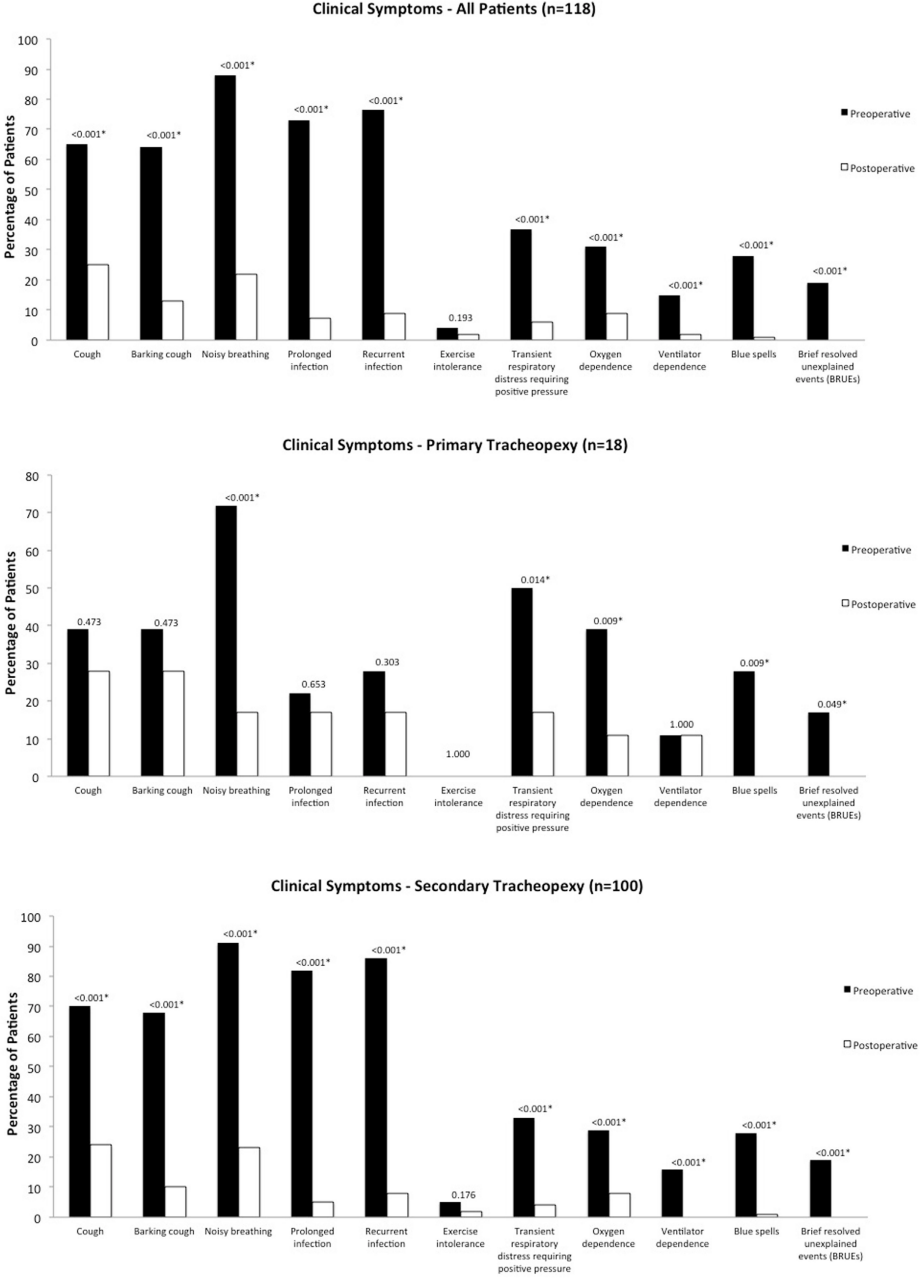
Clinical symptoms. Pre- and postoperative clinical symptoms for the overall cohort, as well as primary and secondary groups.

Median follow-up was 5 months (range 0.25–32 months). Overall, there were statistically significant improvements in clinical symptoms postoperatively, including prevalence of cough, barking cough, noisy breathing, prolonged and recurrent respiratory infections, transient respiratory distress requiring positive pressure, oxygen and ventilator dependence, blue spells, and BRUEs (*p* < 0.001) (Figure 1). There was no significant difference postoperatively in exercise intolerance (*p* = 0.193). At latest follow-up, no patients had recurrence of a BRUE.

For primary cases, there were statistically significant improvements in noisy breathing (*p* < 0.001), transient respiratory distress requiring positive pressure (*p* = 0.014), oxygen dependence and blue spells (*p* = 0.009), and BRUEs (*p* = 0.049). There were no significant differences in cough or barking cough (*p* = 0.473), prolonged respiratory infection (*p* = 0.653), recurrent respiratory infection (*p* = 0.303), and exercise intolerance and ventilator dependence (*p* = 1.000). For secondary cases, there were statistically significant improvements in all clinical symptoms (*p* < 0.001) except for exercise intolerance (*p* = 0.176).

Secondary cases had significantly more baseline preoperative cough (70 vs. 39%, *p* = 0.012), barking cough (68 vs. 39%, *p* = 0.019), prolonged respiratory infections (82 vs. 22%, *p* < 0.001), recurrent respiratory infections (86 vs. 28%, *p* < 0.001), and exercise intolerance (5 vs. 0%, *p* = 0.022) than primary cases, however, there were no significant differences in any postoperative symptoms between the two groups (*p* > 0.1).

In all, 64% underwent postoperative follow-up evaluation with bronchoscopy, 83% of primary patients and 61% of secondary patients. Total tracheomalacia scores on bronchoscopy improved significantly in primary (*p* = 0.013) and secondary (*p* < 0.001) cases, with the greatest areas of numerical improvement in the middle (T2) and lower (T3) trachea (Table 2).

In all, 13% had persistent airway intrusion requiring reoperation, 28% (5/18) in primary cases vs. 10% (10/100) in secondary cases (*p* = 0.05). Reoperations included anterior aortopexy in 9%, anterior tracheopexy in 6%, and revision posterior tracheopexy in 6%. Ten of the reoperations were within 6 months following the index surgery. Kaplan–Meier analysis estimated 88% of patients to be free from reoperation at 6-month follow-up (95% confidence interval 80–96%) and 80% of patients to be free from reoperation at 12-month follow-up (95% confidence interval 70–90%). Multivariable Cox regression analysis indicated that younger patients at tracheopexy (*p* = 0.01) and those with lower preoperative total tracheomalacia scores (*p* = 0.04) were independent risk factors for earlier reoperation, whereas long gap EA (*p* = 0.54) and primary versus secondary EA repair (*p* = 0.14) were not found to be associated with reoperation. There were no significant differences in reoperation between primary and secondary tracheopexy adjusting for imbalances in age and EA type (*p* = 0.67).

Overall, 9% (11/118) had tracheostomies preoperatively, all for severe tracheomalacia in secondary cases. There were no preoperative tracheostomies in primary cases. Overall, 8% (9/118) had tracheostomies postoperatively, with no significant difference when compared to preoperatively (*p* = 0.413). Of the 11 secondary patients (11%) who had preoperative tracheostomies, 7 patients weaned to tracheostomy collar postoperatively and at latest follow-up, 4 patients had undergone tracheostomy decannulation. The two new postoperative tracheostomies were in one primary patient (6%) and one secondary patient (1%), who developed bilateral vocal cord paresis requiring tracheostomy, neither of which had persistent airway intrusion requiring reoperation.

## Discussion

Tracheomalacia is a common respiratory problem among EA patients. Older studies report a prevalence of 11–33% in this population, likely an underestimate given the wide spectrum of disease and common misdiagnosis in the pediatric population, with a recent study reporting tracheomalacia in 87% of EA patients (2, 7–12). Early and accurate diagnosis of tracheomalacia is important because excessive airway collapse leads to ineffective ventilation and poor clearance of secretions, resulting in chronic respiratory symptoms that can progress to bronchiectasis in up to 27% of EA patients by 8 years of age, and in the most severe cases, blue spells and BRUEs (1, 6, 7, 12, 13). The management of severe tracheomalacia remains challenging with little consensus on evaluation, diagnosis, medical treatment, and surgical approach (1–3, 14).

To standardize the diagnosis and treatment of tracheomalacia at our institution, we utilize a standardized scoring system based on anatomic region for endoscopic evaluation (3, 5, 6). The greater the severity of airway collapse, indicated by a lower tracheomalacia score, combined with the presence of clinical symptoms, may warrant surgical correction tailored to the anterior and/or posterior component of tracheomalacia. Surgical options include pexy procedures (ascending and/or descending aortopexy, anterior and/or posterior tracheopexy), tracheal resection, and external stabilization (1–5, 15–17). Anterior ascending aortopexy is the most commonly used technique, but has a reported failure rate of 10–25% in the literature (15–17). Interestingly, posterior tracheopexy to the anterior longitudinal spinal ligament, as first reported by our group, was initially developed in the setting of recurrent TEF repair as an effective strategy to prevent re-recurrence by slightly rotating the tracheal closure to separate it from the esophageal repair (18). However, on follow-up bronchoscopic evaluation, we observed that posterior tracheopexy was quite effective in treating posterior intrusion type tracheomalacia and began using posterior tracheopexy for this purpose. Our initial experience with this technique has shown promising short-term results, however, this series focuses on distinguishing between primary and secondary cases (2, 3, 5).

The majority of our experience with posterior tracheopexy for tracheomalacia has been in secondary cases after prior esophageal surgery (*n* = 100). Secondary cases were mostly patients with prior type C repairs, who underwent bronchoscopic evaluation for chronic respiratory symptoms and were shown to have severe tracheomalacia and low preoperative tracheomalacia scores [median 260 (IQR 216–320)]. Postoperatively, the secondary group showed significant improvements clinically in symptomatology, as well as anatomically in tracheomalacia scores.

More recently, we have used posterior tracheopexy in select primary cases at the time of initial EA repair (*n* = 18), mostly in type A and/or long gap EA. Routine use of preoperative dynamic tracheobronchoscopy as an adjunct to the preoperative assessment of EA is controversial, with only 21.5–60% of surgeons performing preoperative tracheobronchoscopy in this setting (19–21). Associated tracheobronchial anomalies are present in nearly half of EA patients, and endoscopic findings can impact clinical management in 21–45% of patients (22–24). Our multidisciplinary care team routinely uses preoperative dynamic tracheobronchoscopy in all primary EA cases and if severe tracheomalacia is identified, typically defined as coaptation in one or more regions of the trachea, will perform posterior tracheopexy at the time of initial EA repair. The EAT team is only involved in the most difficult EA/TEF cases, while the general pediatric surgery service does not involve the EAT team in 12–15 cases of EA/TEF a year. We rely mainly on bronchoscopic evaluation to determine primary treatment, as clinical symptoms may be confounded in the setting of an unrepaired EA/TEF. There were no significant differences between primary and secondary preoperative tracheomalacia scores, and postoperatively, there were significant clinical and anatomic improvements for primary cases as well.

Posterior tracheopexy is feasible and can be performed at the time of esophageal work. Because we perform posterior tracheopexy on all primary cases with associated coaptation, we do not have a group of primary cases with severe tracheomalacia that are observed to follow its natural history. There is, however, evidence that many children do not outgrow tracheomalacia with up to a quarter of EA patients developing irreversible bronchiectasis by 8 years of age (12). We believe that with an experienced team, posterior tracheopexy can be safely performed at the time of initial EA repair without adding significant additional risk, and may prevent the development of chronic respiratory symptoms and the need for a second operation to correct the tracheomalacia.

Primary tracheopexy in newborns with type C EA, especially at lower birth weights, represents a particularly challenging cohort. We believe that simultaneous flexible bronchoscopy to guide suture placement is critical for success, but performing flexible bronchoscopy with an endotracheal tube (ETT) less than 3.5 can be difficult. Maintaining adequate ventilation during flexible bronchoscopy with a smaller ETT requires an experienced anesthesia team.

Persistent airway intrusion requiring reoperation remains an ongoing challenge, 28% in primary cases and 10% in secondary cases. The majority of reoperations involved additional anterior work, either anterior aortopexy and/or tracheopexy. 8% of secondary cases had already undergone a prior aortopexy, and 20% of overall cases underwent concomitant pexy procedures to further open their airway. In patients with complex heterogeneous tracheomalacia with anterior and posterior components, we favor a flexible and individualized approach tailored to each patient. Our preference is to perform posterior tracheopexy first, as we have found that having the posterior tracheal wall fixed to the spine technically improves the bronchoscopic success of aortopexy, should patients need further anterior work.

Limitations to this study include the retrospective study design. Although patients are followed closely by our multidisciplinary clinic, further studies could utilize a prospective structured clinical symptom questionnaire to further standardize reporting. Bronchoscopy can be subjective and was performed by three primary operating surgeons. One study in adults showed appropriate inter- and intraobserver reliability in flexible bronchoscopy, however, less is known in the pediatric population (25). Postoperative endoscopic evaluation was not available for all patients, however, we used the standardized scoring system to demonstrate resolution of tracheomalacia postoperatively in those evaluated. Our standard protocol for endoscopic postoperative evaluation is at 1 year unless symptomatic. Our study cohort included a heterogeneous group of complex patients requiring adjunct therapies that may have contributed to outcomes and confounded the influence of surgical treatment alone. Follow-up intervals were relatively short term and variable.

In conclusion, posterior tracheopexy is effective in treating severe tracheomalacia associated with EA with significant improvement or resolution of clinical symptoms and degree of airway collapse on bronchoscopy. With no significant differences in outcomes between primary and secondary treatment, posterior tracheopexy should be selectively considered at the time of initial EA repair. A standardized approach to the evaluation of tracheomalacia allows for longitudinal airway assessment and correlation with clinical symptomatology to follow long-term outcomes. Given the heterogeneity and complexity of this patient population with significant morbidity, treatment and long-term follow-up are best done in multidisciplinary EAT centers. Tracheomalacia associated with EA adds a level of complexity that may be best treated with individualized patient care in specialized centers.

## Ethics Statement

This retrospective review was performed at Boston Children’s Hospital under an approved institutional review board protocol (IRB-P00021702).

## Author Contributions

Study conception and design: HS, CS, TH, and RJ. Data acquisition: HS. Analysis and data interpretation: HS, CS, TH, DZ, GV, MM, CB, and RJ. Drafting of the manuscript: HS. Critical revision: HS, CS, TH, DZ, GV, MM, CB, and RJ.

## Conflict of Interest Statement

The authors declare that the research was conducted in the absence of any commercial or financial relationships that could be construed as a potential conflict of interest. The reviewer AC and handling Editor declared their shared affiliation.
